# Integrating longitudinal serum IL-17 and IL-23 follow-up, along with autoantibodies variation, contributes to predict bullous pemphigoid outcome

**DOI:** 10.1038/srep18001

**Published:** 2015-12-14

**Authors:** Julie Plée, Sébastien Le Jan, Jérôme Giustiniani, Coralie Barbe, Pascal Joly, Christophe Bedane, Pierre Vabres, François Truchetet, François Aubin, Frank Antonicelli, Philippe Bernard

**Affiliations:** 1 Department of Dermatology, Reims University Hospital, University of Champagne-Ardenne, Reims, France; 2 Laboratory of Dermatology, Faculty of Medicine of Reims, EA 7319, IFR 53, University of Champagne-Ardenne, Reims, France; 3 Clinical Research Unit, Reims University Hospital, Reims, France; 4 Department of Dermatology, Inserm U905, Institute for Research and Innovation in Biomedicine, Rouen University Hospital, University of Normandy, Rouen, France; 5 Department of Dermatology, Limoges University Hospital, Limoges, France; 6 Department of Dermatology, Dijon University Hospital, Dijon, France; 7 Department of Dermatology, Beauregard Hospital, Thionville, France; 8 Department of Dermatology, Besançon University Hospital, Besançon, France

## Abstract

Bullous pemphigoid (BP) is an inflammatory autoimmune bullous disease involving cytokines and proteases in the process of blister formation. Recently, IL-17 and IL-23 were evidenced in lesional skin and serum of BP patients at time of diagnosis, but their involvement in disease outcome has still not been investigated yet. We then analysed IL-17 and IL-23 serum levels during the first months of follow-up upon treatment. Compared with age- and sex- matched controls, high levels of IL-23 were observed at baseline in BP patients serum (*P* < 0.01), while IL-17 levels was not. However, some BP patients expressed high IL-17 serum level, independently of disease severity. In these patients, those with ongoing remission reduced IL-17 concentration upon treatment (*P* < 0.001), whereas IL-17 level remained elevated in patients who relapsed. Meanwhile, IL-23 serum levels increased during the first month of treatment in BP patients who later relapsed (*P* < 0.01) and MMP-9 serum level was not controlled. Accordingly, we found that both IL-17 and IL-23 increased MMP-9 secretion from leukocytes *in-vitro*. Then, we showed that elevated IL-17/IL-23 serum concentrations helped to discriminate BP patients who later relapsed. Such uncontrolled inflammatory response raises the question whether these molecules could become biological target for BP patients resistant to steroid treatment.

Bullous pemphigoid (BP) is the most frequent auto-immune blistering disease, representing 70% of the subepidermal bullous disease subgroup, and preferentially affects the elderly^1^,^2^,^3^. It is clinically characterized by tense blisters, occurring on inflammatory erythematous plaques with intense itching^2^,^3^,^4^. The inflammatory clinical presentation of BP has supported its treatment with superpotent topical corticosteroids (CS), which is currently the first line of treatment in France^5^,^6^. While CS treatment enables sustained remission in the majority of patients^6^,^7^, BP relapse rate remains significant (around 30%) within the first year of treatment^1^. Those relapses have been associated with disease extent at baseline^8^, but the underlying biological pathophysiological mechanisms allowing the identification of patients that relapse still needs to be deepened.

Immunologically, BP is characterized by the production of autoantibodies directed against two major components of the hemidesmosome, BP180 and BP230^9^,^10^. Autoantibody antigen binding^11^ causes an inflammatory cascade response at the dermal-epidermal junction leading to the secretion of a variety of inflammatory mediators resulting in overexpression of proteases such as matrix–metalloproteinases-9 (MMP-9) and neutrophil elastase^12^,^13^ responsible for blister formation. In a recent study, we found that blister fluids displayed high levels of IL-6, IL-17, IL-22, and IL-23, although we could not confirm any Th17 lymphocytes orientation as previously proposed^14^,^15^,^16^,^17^, but highlighted an association between IL-17 level and protease overexpression. Therefore, these results highly suggested the possible involvement of IL-17 in the pathogenesis of BP^17^.

IL-17 has been implicated as a major pathogenic factor in chronic inflammatory diseases as well as in autoimmune diseases such as rheumatoid arthritis, inflammatory bowel disease, psoriasis^18^ by recruiting in lesional tissue polymorphonuclear neutrophils (PMN) and eosinophils, which have been shown to be key inflammatory cells in the pathogenesis of BP^19^,^20^. Interestingly, IL-17 is not only produced by Th17 cells, but also by a variety of innate cells such as macrophages, dendritic cells and neutrophils, notably in the skin of BP patients^17^,^21^. Several pathways have been proposed for the overexpression of IL-17, but IL-23 remains the most important molecule mediating IL-17 expression whatever the cell type^22^.

In a previous study, we demonstrated the usefulness of monitoring anti-BP180 autoantibodies, and its clinical value as a potential risk factor for BP relapse during the first year of treatment^8^. In this ancillary study, we monitored the serum level of IL-17 and IL-23 during the course of the disease on a large series of BP patients. We investigated whether the serum concentration of these cytokines and of the protease MMP-9 varied according to initial disease extent or to BP relapse. Finally, we wondered whether IL17 and IL-23 could also enhance the release of MMP-9 from blood cells and therefore actively participate to the inflammatory response.

## Results

### Characteristics of patients

One-hundred and twenty patients were included between November 2009 and May 2012. The mean age at diagnosis was 81.1 years and the sex ratio F/M was 1.35. Among these 120 patients, 53 (44%) had extensive disease (i.e. more than 10 daily blisters). During the 1-year follow-up, 25 patients (21%) died, 7 (6%) were lost to follow-up and 13 (10%) dropped out of the study. All the living patients at month 2 (108 out of 120) achieved disease control, and 35 patients (29.2%) experienced at least one relapse then after this initial disease control within a follow-up of 1 year.

### Cytokine serum levels at baseline

**Figure 1:**
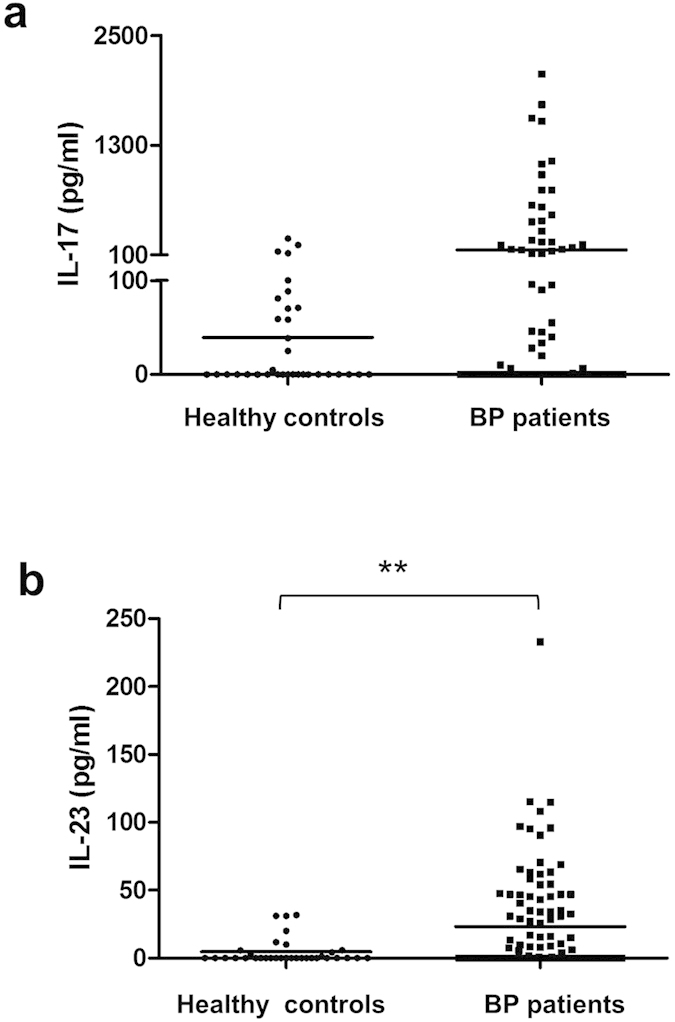
Variations of IL-17 and IL-23 levels in BP patients compared to healthy controls. Serum concentrations of IL-17 (**a**) and IL-23 (**b**) were measured by ELISA in BP patients at time of diagnosis and in healthy controls. (**P  < 0.01).

Serum measurements of IL-17 and IL-23 were performed at baseline in the 120 BP patients and in 34 healthy controls (Fig. 1). At baseline, IL-17 serum concentrations were not statistically different between BP patients and controls (158±36 *vs* 40 ± 11 pg/mL, respectively), whereas IL-23 levels were significantly more elevated in the serum from BP patients than in controls (23±4 *vs* 5±2 pg/mL, respectively; *P* < 0.01). At baseline, neither IL-17 nor IL-23 concentration varied according to disease extent (Table 1A). Also, the serum levels of these cytokines at baseline did not allow discriminating BP patients that relapsed during the first year of treatment from patients with ongoing remission (Table 1B). Nevertheless, a non-Gaussian distribution of serum IL-17 levels in BP population was demonstrated by a normality test. Indeed, 71 patients (64%) did not express IL-17, 13 (12%) had low IL-17 level similar to healthy donors, and 26 (24%) expressed high level of IL-17. However, such distribution was still not significantly associated to BP extent.

### Serum cytokine variations between baseline and day 150 and clinical outcome

A longitudinal study was then undertaken to analyse IL-17 and IL-23 variations in the serum of BP patients under treatment. The analysis was performed only in patients for whom serum samples were available both at day 0, day 60 and day 150 (n= 60 for IL-23 and n=56 for IL-17). The clinical characteristics of this subgroup of BP patients were similar to the whole study population in terms of sex ratio, mean age, percentage of patients with extensive *versus* moderate disease, and rate of BP relapse within the first year of treatment (data not shown). All these patients achieved disease control between day 0 and day 60.

**Figure 2:**
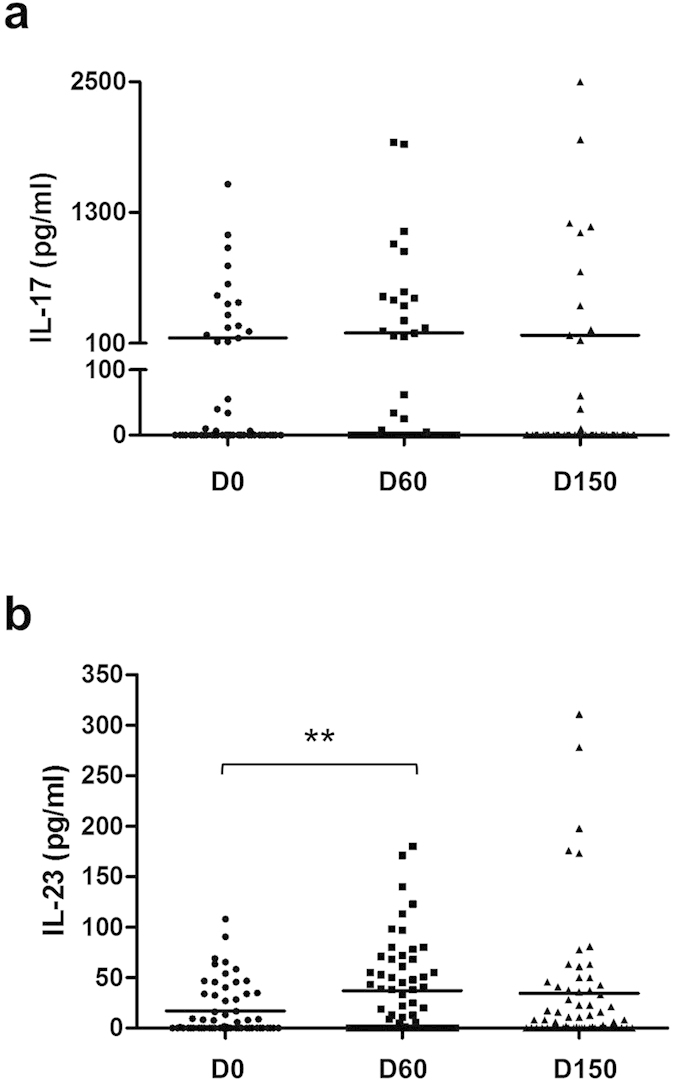
Variations of IL-17 and IL-23 concentration in BP serum from baseline until day 150. Serum concentrations of IL-17 (**a**) and IL-23 (**b**) were measured by ELISA at time of diagnosis, 60 days and 150 days after the inclusion of BP patients. (**P  < 0.01) Abbreviations: D0, day 0; D60, day 60; D150, day 150.

The longitudinal serum cytokine analysis showed no significant variation of IL-17 serum concentrations within the study period (Fig. 2a). Of note, the non-Gaussian distribution of IL-17 values was still observed at day 60 and day 150, with a group of BP patients that expressed high serum level of this cytokine. Meanwhile, serum concentrations of IL-23 significantly increased from 17 ± 3 pg/mL to 37 ± 6 pg/mL (*P* < 0.01) between day 0 and day 60, and remained elevated thereafter between day 60 and day 150 (Fig. 2b).

**Figure 3:**
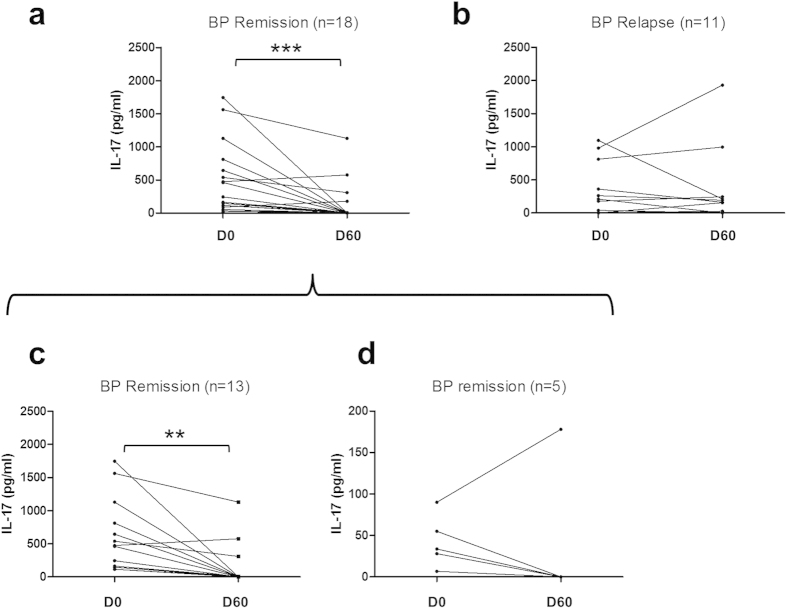
Variations of IL-17 concentrations in BP serum from baseline until day 60 according to later BP relapse. Serum concentrations of IL-17 were measured by ELISA at time of diagnosis and at day 60 in patients who were in remission (**a**) and patients who further relapsed (**b**). The subgroup of patients who expressed IL-17 at diagnosis (**a**) was analysed and divided into 2 subgroups: the first one for which IL-17 concentration was higher than 100 pg/ml at diagnosis (**c**) and the second one for which IL-17 concentration was higher than 0 but lower than 100 pg/ml (**d**). (**P < 0.01; ***P < 0.001)

Then, we wondered whether IL-17 and IL-23 cytokine concentration variations within the 2 first months of follow-up could discriminate BP patients who relapsed within the first year of treatment from those who remained on remission (Fig. 3, Table 2). We performed the analysis in those BP patients who expressed detectable IL-17 serum levels at baseline. At time of diagnostic, IL-17 serum levels were not informative of BP outcome (Table 2). However upon treatment, IL-17 level only decreased significantly between D0 and D60 in the subgroup of BP patients that were controlled up to one year (*p*  < 0.001) (Fig. 3a,b, Table 2). Such a decrease in IL-17 serum level was observed in both BP patients subpopulations with ongoing remission, the one with high level of IL-17 at baseline (*p*  < 0.001) (Fig. 3c), and the one with IL-17 concentration in the range of the healthy controls (4 out of 5 patients) (Fig. 3d). In contrast, IL-17 serum levels remained stable over the 2 first months within the subgroup of BP patients that will later relapse (Fig. 3b).

**Figure 4:**
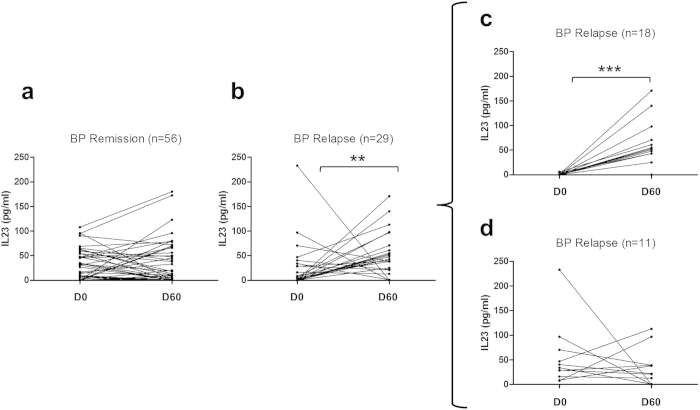
Variations of IL-23 concentrations in BP serum from baseline until day 60 according to later BP relapse. Serum concentrations of IL-23 were measured by ELISA at time of diagnosis and at day 60 in patients in remission (**a**) and patients who later relapsed (**b**). The group of patients who further relapsed was divided into 2 subgroups according to IL-23 concentrations at diagnosis: in serum in which IL-23 was undetectable at time of diagnosis (**c**), and in serum in which IL-23 was detectable at diagnosis (**d**). (**P < 0.01; ***P < 0.001)

Serum IL-23 concentrations were significantly higher (*P* < 0.01) at day 60 compared to day 0 in patients who later relapsed, while no significant variation was observed in those with ongoing remission (Fig. 4a,b). Of note, IL-23 concentration increase was only evidenced in BP patients with low level of IL-23 at baseline (n=18, *P*  < 0.001) (Fig. 4c,d). In this subgroup, the mean IL-23 concentrations varied from 0.6±0.4 to 50.9±11.3 pg/mL. As IL-23 was the only cytokine that significantly increased at baseline compared with controls and between day 0 and day 60 in BP patients who later relapsed, we determined the predictive values relative to this increase. The increase of IL-23 serum concentrations between day 0 and day 60 showed a sensitivity of 76.2% [58.0–94.4], a specificity of 33.9% [21.5–46.3], a negative predictive value of 79.2% [62.9–95.4] and a positive predictive value of 30.2% [17.8–42.5] for BP relapse during the first year of treatment.

### Serum cytokines profiles and MMP-9 secretion

**Figure 5:**
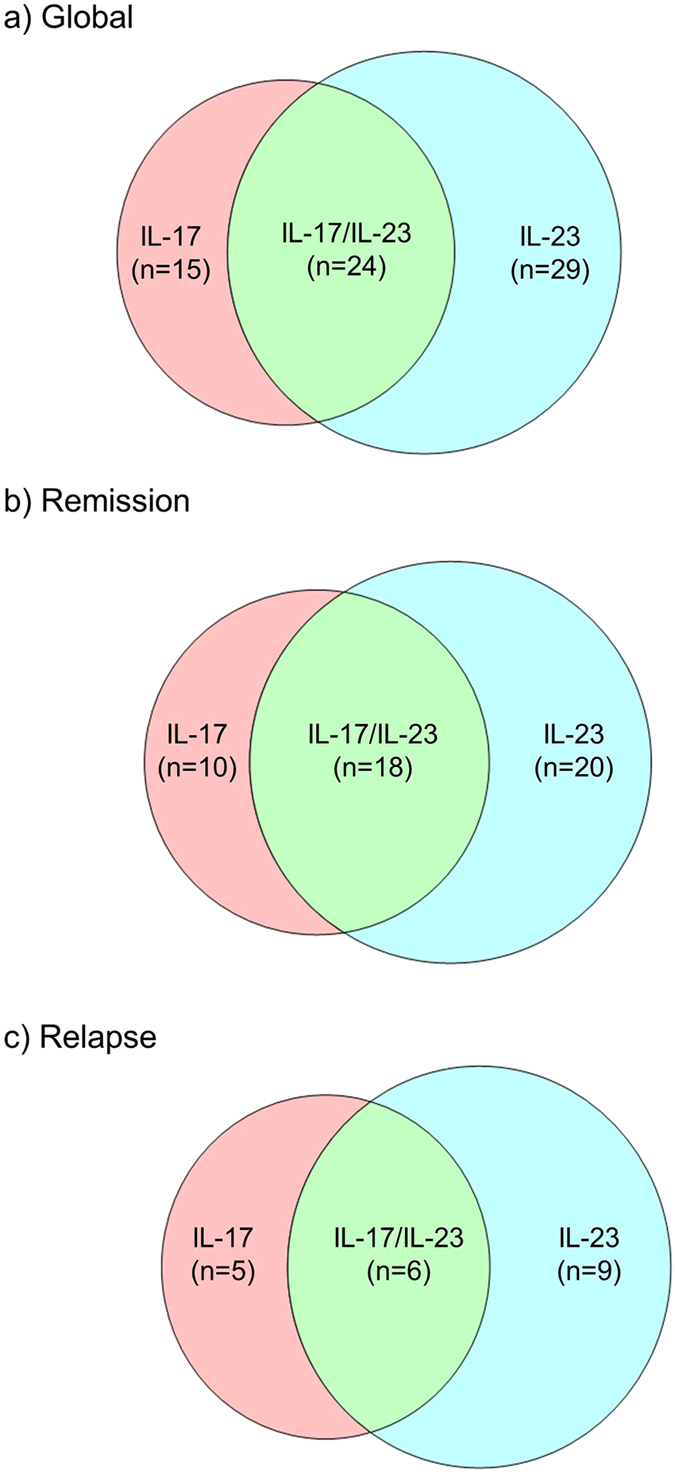
Distribution of IL-17 and IL-23 expression in BP populations. Venn diagram showing the number of patients that express IL-17 alone, IL-23 alone and both IL-17 and IL-23 at diagnosis in global BP population (**a**), and in the population of BP patients who were controlled under therapy (**b**) and who further relapsed (**c**).

**Figure 6:**
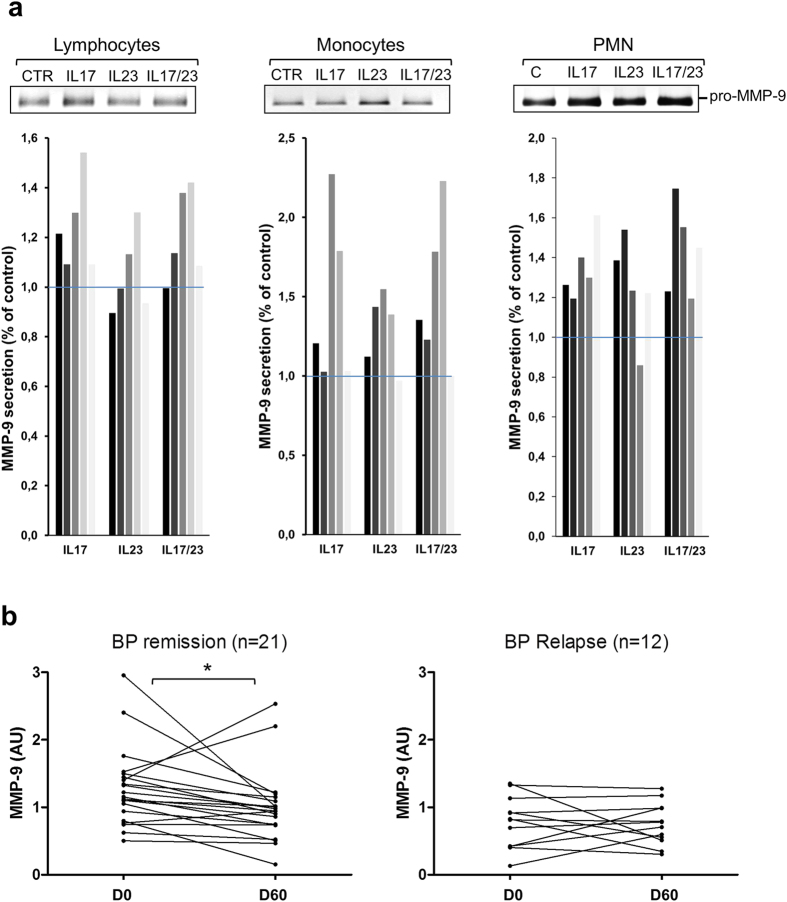
MMP-9 secretion is regulated by IL-17 and IL-23 and varied along the treatment. Lymphocytes, monocytes and polymorphonuclear cells (PMN) were isolated from 5 healthy controls and stimulated with IL-17 (200 pg/ml), IL-23 (100 pg/ml) or both cytokines for 20 hours (mononuclear cells) or 1 hour (PMN). MMP-9 secretion was analysed by gel zymography. One representative zymogram was shown for each type of cells and quantification of zymogram was performed using ImageJ software (**a**). MMP-9 secretion was analysed by gel zymography in BP patients in remission or with relapse at diagnosis and at day 60 (**b**).

BP relapse is characterized by the occurrence of new blisters, which are the result of the dermal-epidermal cleavage upon the action of proteases such as MMP-9. Using blood cell from healthy donors, we investigated whether both IL-17 and IL-23 either alone or combined could increase the secretion of MMP-9, as we observed that approximately 22% of the patients expressed IL-17 alone, 43% expressed only IL-23 and 35% expressed both cytokines, and this whatever the progression of the disease (Fig. 5). MMP-9 secretion was increased by both IL-17 and IL-23 from lymphocytes (29% and 14%, respectively), monocytes (57% and 38%, respectively) and PMN (48% and 26%, respectively) (Fig. 6a). However, IL-17 effects on MMP-9 secretion were more prominent in lymphocytes, while IL-23 preferentially enhanced MMP-9 from monocytes. Interestingly, combined stimulation with both IL-17 and IL-23 did not provide any cumulative or synergistic effects whatever the cell type.

Analysis of concentrations variation of this protease in the serum of BP patients during the initial phase of treatment showed that MMP-9 level decreased between day 0 and day 60 in the whole study population (−0.15 AU; *P* =< 0.05). In accordance with the above results, MMP-9 serum level significantly decreased in patients with ongoing remission (−0.20 AU; *P* < 0.05) but not in the serum of patients that relapsed during the first year of follow-up (Fig. 6b).

## Discussion

In a recent study, we documented the potential role of IL-17 on protease expression at time of diagnosis and therefore in BP blister formation^17^. In this longitudinal study, we showed that both IL-17 and IL-23 serum level varied differently in patients who relapsed compared to patients with ongoing remission during the first year of treatment. Furthermore, we showed that IL-23 also increased MMP-9 secretion from blood cells. To the best of our knowledge, our present study is the first to link during disease monitoring the IL-17/IL-23- inflammatory cytokines secretion to BP outcome.

We reported here that although not statistically different from healthy controls, the serum level of IL-17 was not evenly distributed within the population of BP patients. Indeed, only 35% of these patients expressed detectable level of this cytokine in their serum. Similarly, it has been shown that serum IL-17 levels in patients affected with psoriasis was not different from controls, except in those with the pustular subtype that expressed high levels of IL-17^23^. However, conversely to psoriatic patients, IL-17 serum level in BP patients was not associated to disease extent. Furthermore, the absence of IL-17 in the serum (64%) or in the blister fluid (30%) of BP patients suggests that this cytokine is more likely to originate from an epiphenomenon that could interfere in disease outcome than an inherent molecular intermediate involved in BP pathophysiology. One attractive possibility is that presence of IL-17 could be driven by alteration of skin microbiota as demonstrated in atopic dermatitis^24^, and therefore would be patient dependent.

In contrast to IL-17, IL-23 level was increased in the serum of BP patients with respect to sex- and age-matched controls. Intriguingly, we did not confirm our result obtained in a previous study performed on a smaller population in which IL-23 serum concentration in BP patients and in control were not statistically different^17^. As all statistical analyses have been performed with the same tools, such discrepancies could be linked to the size of the two populations analyzed (patients and controls) which were twice as large as in the previous study. In this line, it is also possible that enlargement of the studied population unmasked a subgroup of patients since, only half of the BP serum analyzed displayed positive values for IL-23 as mentioned for IL-17 above. Besides, the fact that compared with IL-17 values, positive IL-23 serum concentrations were more evenly distributed could also interfere on the statistical analysis.

Moreover, we found that serum IL-23 concentration further increased within the first two months of treatment in BP patients who later relapsed, although they all were clinically controlled during this period of time. Noteworthy, control of the disease has been defined by the international panel of experts as the beginning of the consolidation phase, and therefore after 2 months of treatment BP patients were controlled but not cured yet^25^. Furthermore, IL-23 variations during this period of time are in setting with our previous publication showing that although a more pronounced decrease of serum anti-BP180 autoantibodies was observed between baseline and day 60 in patients with ongoing remission than in patients with relapse, the autoantibody level remained elevated after 2 months of treatment^8^. Then an implicit pathologic process must persist overtime in most BP patients. In the present study, we highlighted the fact that the level of IL-23 could be one of the molecules associated to this underlying immunological process. Indeed, we showed that IL-23, like IL-17 increase could participate to the ongoing inflammatory process by increasing MMP-9 secretion, one of the main proteases involved in blister formation. This is of interest as we previously showed that BP is not a Th17 related disease^17^, and therefore that IL-23 related mechanisms might dissociate at some stage from those of IL-17.

Serum IL-23 increase at day 60 was mainly observed in BP patients with null or very low level of this cytokine at baseline. Only few molecules, especially bacterial products and prostaglandins have been shown to increase IL-23 secretion, and little is known about the cellular sources implicated in the generation of IL-23^26^,^27^. Several recent studies have identified monocytes, macrophages and dendritic cells as a source of IL-23^27^,^28^,^29^,^30^. Also, a cutting edge publication showed that IL-23 was secreted only by monocytes and not lymphocytes issued from blood of patients with autoimmune celiac disease^31^. Although further studies are required to unravel the stimuli and the cells involved in IL-23 expression in BP, it is possible that IL-23 production could also be associated to an uncontrolled skin microbiota as suggested above. In this setting, it will be of interest to investigate whether the observed serum IL-23 overproduction occurs in the blood or if it is representative of an *in situ* host response.

It is supposed that in BP the autoimmune response creates an inflammatory environment that can refuel the autoimmune process leading to disease progression or persistence. In many diseases, members of the IL-17 family participate to inflammation by enhancing cytokines, chemokines and MMPs secretion leading to tissue damages^32^,^33^,^34^,^35^. But conversely to BP, expression IL-17 and IL-23 in those diseases have not been associated to subsequent blister formation. Of note, most of these inflammatory responses were associated to the Th17 lineage. In contrast, we previously illustrated that in BP, IL-17 was mainly produced by PMN and mast cells^17^ suggesting that blister formation is associated to specific pathophysiological processes. Besides, it has been shown recently in an elegant study that replacement of IL-4 and IL-13 by IL-17 in a combination of several cytokines led to the switch from an atopic dermatitis-like to a psoriasis-like three dimensional *in vitro* model^36^. Then, cellular and molecular environments in the vicinity of BP skin lesions could orientate IL-17 and IL-23 function specificities. In BP, such specificity could be related to the autoimmune response directed against the auto-antigenic proteins BP180 and BP230, which focuses the inflammatory reaction towards the hemidesmosomal structure.

Interestingly, the population of BP patients who expressed IL-17 at baseline was partially distinct from the BP population expressing IL-23, suggesting that both cytokines may independently participate to BP outcome. In the present study, we found that the subgroup of BP patients that later relapsed displayed sustained serum MMP-9 level, whereas it decreased over time in the serum of patients with ongoing remission. Similarly to MMP-9, IL-17 and IL-23 serum levels remained elevated in BP patients who relapsed during the first year of treatment. We previously published that IL-17 played a critical role in the pathophysiology of BP by increasing both MMP-9 from PBMC and PMN^17^. In this study, we showed that MMP-9 secretion was also under the control of IL-23, which is a new protagonist in the protease activity regulation associated to BP. Furthermore, our study showed for the first time that stimulated human monocytes also released MMP-9. Although stimulated PMN and lymphocytes can directly participate to blister formation by releasing proteases at site of lesion, monocytes are found in the blood stream or stored in lymphoid tissue. However, monocytes can quickly migrate to lesional skin and divide/differentiate into macrophages and dendritic cells to elicit an immune response. Therefore, increase of MMP-9 secretion from monocyte upon IL-7 and IL-23 stimulation could facilitate the extravasation and migration of monocyte cells before they differentiate into macrophages, which have been associated to the pathological process of BP^37^,^38^. Subsequently, *in situ* MMP-9 released by all these cell types could degrade matrix molecules into peptides that could feed back the inflammatory and immune responses as described in our previous study^17^.

Besides this direct role on MMP-9 production, IL-17 and IL-23 serum concentration could also influence the outcome of BP patients under therapy by interfering with treatment responsiveness. Indeed, it was recently shown in human bronchial epithelial cells that IL-17 modulated the epigenetic mechanisms involved in the transcriptional activity of glucocorticoid receptors^39^. Furthermore, it has recently been shown an up-regulation by IL-17/IL-23 of glucocorticoid receptor-beta, which is associated with corticosteroid resistance, leading to steroid insensitivity in PBMCs^40^. Therefore, it would be of interest in the near future to investigate whether such relationship between IL-17, IL-23 and CS insensitivity could be also related to BP relapse.

Based on our above-mentioned hypothesis, an insufficient therapeutic response to CS, which would not control enough the systemic inflammatory response, could favour BP relapses. Although measuring serum anti-BP180 autoantibodies several months after starting BP therapy is of interest^8^, this might be too restricting with regards to the inflammatory status of BP patients. Indeed, patients at high risk of BP relapse generally require additional immunomodulatory therapeutic agents as in chronic inflammatory diseases such as psoriasis, Crohn’s disease or rheumatoid arthritis^18^. As an alternative therapy, low-dose methotrexate has been proposed for long-term maintenance therapy in BP after clinical remission obtained by initial, short-term treatment with superpotent CS^41^. However, like classical immunosuppressant agents, methotrexate has a number of side effects or contraindications in elderly patients^41^. Therefore, biologics that target components of the IL-23/IL-17 cascade may represent new therapeutic horizons for BP with a better satisfactory benefit/risk ratio than immunosuppressants, similarly to psoriasis where serum IL-17 and IL-23 levels are increased at baseline and correlated with disease severity^42^. In this setting, several biologics, including ustekinumab, a monoclonal antibody that targets the p40 subunit of IL-12/23^43^,^44^,^45^ or monoclonal antibodies targeting IL-17 or its receptor such as secukinumab, ixekizumab and brodalumab^46^ represent potential candidates for maintenance therapy in BP patients by allowing the reduction of the cumulative doses of CS by rapidly tapering and stopping them after disease control.

In a previous study, we investigated the autoimmune response associated to BP and demonstrated the interest of measuring BP autoantibodies during BP patients follow-up. The present study has deepened our understanding of the inflammatory response in BP and its likely implication in patients at risk for disease relapse. It also demonstrates that BP progression is not driven by only one key molecule, and that a panel of inflammatory molecules constituting an “inflammascore” could be useful, in combination with the determination of specific BP autoantibodies, to predict the likelihood of relapse.

## Methods

### Study Patients and Design

This prospective, multicenter, observational study was conducted in 8 French dermatology departments among which 3 belong to the French Referral Center for Autoimmune Bullous Diseases (Reims, Rouen, Limoges). Inclusion criteria were consecutive patients with newly diagnosed BP using the following criteria: clinical features typical of BP with presence of at least three out of four well-established criteria by Vaillant *et al.*^47^; subepidermal blister on skin biopsy; and deposits of IgG and/or C3 in a linear pattern along the epidermal basement membrane zone by direct IF. All voluntary subjects gave their written informed consent before inclusion in the study. Control sera were obtained from age- and sex-matched patients without inflammatory and autoimmune diseases hospitalized in the department of traumatology and orthopedic surgery of the same hospital

### Ethics statement

The study was approved by the Ethics Committee of the University Hospital of Reims (institutional review board; 14.04.2009); patients or their relatives were informed by letter and gave their written consent, in accordance with the Helsinki Declaration.

### Baseline and follow-up measurements

#### Clinical data

BP patients were followed for 1 year for clinical outcome assessment with 6 visits planned as previously described^8^. Baseline clinical evaluation included the number of daily new blisters for 3 consecutive days, localization of skin or mucous membrane blisters and erosion. Extensive BP was defined as the occurrence of at least 10 daily new blisters; otherwise BP was classified as moderate^6^,^7^. At each visit, the occurrence and date of relapse which may have occurred, or the date of early study release and its cause (death, lost to follow-up, serious adverse reaction, other reason) were recorded. Relapse was defined as the reappearance of at least 3 new daily blisters along with pruritus and/or erythematous, eczematous or urticarial plaques^4^.

#### Serum cytokine measurement

Blood samples were collected at baseline, then at days 60 and 150 for serum measurement of IL-17 and IL-23. Collected blood samples were centrifuged for 15 minutes at 1000 × g. Serum aliquots were stored at −80 °C until being tested for cytokine concentration using specific ELISA Kits with respect of the instructions provided by the manufacturer (R&D Systems). Cytokine concentration was determined using 100 μL of serum for each ELISA, by reference to the standards provided within the kits. Results were expressed in picomol/milliliter. According to the manufacturer, there was no significant cross-reactivity or interference with other known cytokines in these assays.

#### Gelatine zymography

Quantity of pro-MMP-9 was determined using 2 μL of serum from BP patients by the zymography technique. Briefly, serum samples were electrophoresed on a 10% SDS polyacrylamide gel impregnated with 0.1% of gelatin under non-reducing conditions. After migration, proteinases were re-activated within the gel by incubation at 37 °C overnight in a buffer containing 50 mM Tris-HCl (pH 7.5), 10 mM CaCl2 and 0.02 mM NaN3. The gel was subsequently fixed and stained with 0.25% Coomassie brilliant blue R-250 for 1 h and washed in 25% methanol and 7% acetic acid to visualize the proteolytic activity bands. Data were expressed by converting the optical density in arbitrary units (AU) with the software Image J.

### *In vitro* blood cell stimulation

Peripheral Blood Mononuclear Cells (PBMCs) and PMN from healthy donors were obtained by density-gradient centrifugation from EDTA-treated whole blood using a density gradient medium (Granulosep, Eurobio-Abcys). Monocytes were purified from PBMCs by positive selection using CD14 immunomagnetic beads (MACS; Miltenyi Biotech) according to manufacturer instructions. Flow-through was then used to isolate T-lymphocyte by negative selection using the Pan T-cells isolation kit from Miltenyi (MACS).

Isolated leukocytes from healthy donors were then cultured in FBS-free medium and stimulated overnight (lymphocytes and monocytes) or for 1 hour (PMN) by IL-17 (200 pg/mL), IL-23 (100 pg/mL), or both cytokines. Culture media were harvested and analyzed for MMP-9 secretion by gel zymography.

### Statistical analysis

Quantitative variables were described as means ± standard deviation (SD) and qualitative data as number and percentage. Value distribution was analysed using D’Agostino and Pearson omnibus normality test. Comparisons among groups were performed by Mann Whitney test. Correlations among cytokines and among cytokines and anti-BP180 autoantibodies were performed by Pearson correlation coefficient test. Cytokines and proMMP9 values variations during follow-up were compared according to the occurrence of cutaneous relapses with Wilcoxon test. Whatever the test used, a *P*-value < 0.05 was considered significant. All statistical analyses were performed using Prism® software.

## Additional Information

**How to cite this article**: Plée, J. *et al.* Integrating longitudinal serum IL-17 and IL-23 follow-up, along with autoantibodies variation, contributes to predict bullous pemphigoid outcome. *Sci. Rep.* **5**, 18001; doi: 10.1038/srep18001 (2015).

## Figures and Tables

**Table 1:**
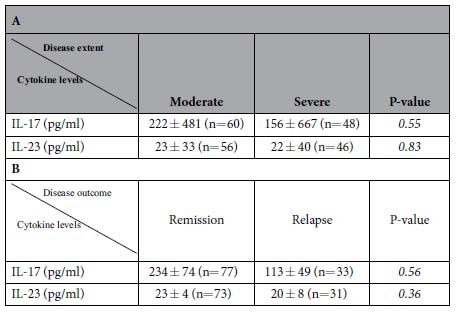
IL-17 and IL-23 levels in BP patients at diagnosis according to the extent (A) or to the outcome of the disease (B). Extensive disease was defined as more than 10 new blister daily.

Extensive disease was defined as more than 10 new blister daily.

**Table 2:**
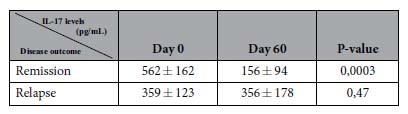
Effects of treatment on IL-17 serum levels in BP patients with [IL-17] > 0 according to their clinical outcome (remission or relapse).
